# Transcranial Direct Current Stimulation (tDCS) as a Useful Rehabilitation Strategy to Improve Cognition in Patients With Alzheimer's Disease and Parkinson's Disease: An Updated Systematic Review of Randomized Controlled Trials

**DOI:** 10.3389/fneur.2021.798191

**Published:** 2022-02-02

**Authors:** Davide Maria Cammisuli, Fabio Cignoni, Roberto Ceravolo, Ubaldo Bonuccelli, Gianluca Castelnuovo

**Affiliations:** ^1^Department of Psychology, Catholic University of the Sacred Heart, Milan, Italy; ^2^Neurological Clinic, Department of Clinical and Experimental Medicine, University of Pisa, Pisa, Italy; ^3^Department of Clinical and Experimental Medicine, University of Pisa, Pisa, Italy; ^4^Azienda Unità Sanitaria Locale (USL) Toscana Nord Ovest, Pisa, Italy; ^5^Psychology Research Laboratory, Istituto Auxologico Italiano IRCCS, Milan, Italy

**Keywords:** transcranial direct current stimulation, cognition, rehabilitation, randomized controlled trials, Alzheimer's disease, Parkinson's disease

## Abstract

Alzheimer's disease (AD) and Parkinson's disease (PD) are neurodegenerative disorders characterized by cognitive impairment and functional decline increasing with disease progression. Within non-pharmacological interventions, transcranial direct current stimulation (tDCS) might represent a cost-effective rehabilitation strategy to implement cognitive abilities with positive implications for functional autonomy and quality-of-life of patients. Our systematic review aimed at evaluating the effects of tDCS upon cognition in people suffering from AD and PD. We searched for randomized controlled trials (RCTs) into PubMed, Web of Science, and Cochrane Library. Three review authors extracted data of interest, with neuropsychological tests or experimental cognitive tasks scores as outcome measures. A total of 17 RCTs (10 trials for AD and 7 trials for PD) were included. Compared with sham stimulation, tDCS may improve global cognition and recognition memory in patients with AD and also some executive functions (i.e., divided attention, verbal fluency, and reduction of sensitivity to interference) in patients with PD. Criticism remains about benefits for the other investigated cognitive domains. Despite preliminary emerging evidences, larger RCTs with common neuropsychological measures and long-term follow-ups establishing longevity of the observed effects are necessary for future research in applied psychology field, alongside improved clinical guidelines on the neurodegenerative disorders pertaining electrodes montage, sessions number, duration and intensity of the stimulation, and cognitive battery to be used.

## Introduction

### Application of Transcranial Direct Current Stimulation (tDCS) in Cognitive Rehabilitation

The tDCS is a neurostimulation method, painless, substantially devoid of the significant side effects, economic, simple to apply and even suitable for a home environment administration under supervision of remote therapist, also in case of the neurological disorders (1–4). In such a technique, a weak current—usually 1/2 mA at constant frequency—is applied to the scalp through one or two stimulation electrodes in targeted brain regions, as single or bilateral configuration modes (5). The current leads to changes in the extracellular *milieu* that, in turn, affects the resting membrane potential of the neuronal populations in the proximity of electrodes placement (6). However, although stimulation is applied over limited brain areas, the distribution of the current that reaches the cortex depends on intensity, modulation duration, electrodes montage and size, and orientation of the electric field in relation to anatomical features of the cortex (7). While the anodal tDCS increases cortical excitability in the brain region under and around the electrode placement, the cathode tDCS decreases it (8). Short-term effects of the tDCS occur through non-synaptic mechanisms by depolarization of resting membrane potential, while long-term effects likely occur through NMDA-dependent mechanisms and appear to be consistent with synaptic plasticity (9, 10).

Despite potential associated adverse events (e.g., tingling, itching, burning sensation, mild headache, bright flashes of light and skin burn, etc.) (6), tDCS is globally considered as a safe, tolerable and low-cost rehabilitation strategy. Contraindications only pertain to metallic implants in the head/body, craniotomy or history of seizure (11, 12). As a result, tDCS has been applied with promising results to many neurological disorders (13–16) and neuropsychiatric conditions (17–20), resulting in an exponential growth of studies in the last decades.

### Cognitive Deficits in Alzheimer's Disease and Parkinson's Disease

Recently, the development of novel non-invasive methods of brain stimulation, such as transcranial magnetic stimulation (TMS) and tDCS has increased the interest in neuromodulatory approaches as potential tools to counteract a progressively more severe cognitive deterioration related to the course of neurodegenerative disorders, such as AD and PD.

On one side, AD is a progressive neurodegenerative disorder and accounts for most of dementia in elderly people, currently affecting 5.8 million people in the US alone (21). This percentage is dramatically estimated to increase, by reaching 65.7 million people affected by AD in 2030 worldwide (22). The number of new cases of AD significantly increases with aging, with an incidence of 76 of every 1,000 people of 85 years and older (21). AD has a devastating effect on patients and their caregivers and determines a tremendous socioeconomic impact on the health system. Usually, cognitive deficits are present in patients prior to the time of AD onset (i.e., mild cognitive impairment due to AD) (23), and typically affect episodic memory and executive functions domains (24). Usually, memory impairment is the earliest representing the core symptom of the disease and functional autonomy of patients decreases with progression of AD, also as a consequence of a wider range of supplementary cognitive deficits (25). Cholinesterase inhibitors are considered as the main pharmacologic treatment for patients with AD although response is quite limited (26–28).

On the other side, PD represents another chronic neurodegenerative disorder leading to a progressive decrement of functional autonomy of the patients. It affects about 1% of people who are aged older than 60 years and reported standardized incidence rates of PD are 8–18 per 100.000 person-years (29). Because of the dopamine reduction in the *pars compacta* of the *substantia nigra*, typical motor symptoms are characterized by resting tremor, rigidity, bradykinesia, and postural instability. Patients with PD show additional motor deficits including gait disturbance and motor complications, such as dyskinesia in the course of the disease (30). Despite its nosographic definition remarking motor deficits, PD has been progressively conceived as a “complex brain disease” including non-motor symptoms, such as cognitive disturbances (31, 32). In PD, there is a *spectrum* of cognitive dysfunction, ranging from mild cognitive impairment (PD-MCI) to PD dementia (PDD). Cognitive impairment is quite common in PD, affecting approximately 30–40% of the patients (33). Cognitive deficits might be present at early stages of the disease and are usually characterized by executive functions and visuospatial deficits (34–36). Neurocognitive deterioration pertaining to the frontal domains and attention system is a consequence of dopamine reduction (i.e., frontal-striatal syndrome). Structural abnormalities of fronto-parietal areas and subcortical regions (37) and temporo-parietal regions (38) implicated in visual stimuli analysis have been observed in patients with PD, too. A particular impairment of implicit motor sequence learning (IMSL) is also displayed by patients with PD, consisting of difficulties in acquisition of multiple single movements to be performed in a sequential order without conscious awareness needed for retrieval (39).

Alternative non-invasive neurostimulatory techniques such as tDCS require urgent development in the next future, both for AD and PD. However, performed investigations on tDCS effects upon cognition in patients with AD and PD to date present some limitations. They did not focus only on randomized controlled trials (RCTs) (22, 40, 41), mixed results from TMS and tDCS (42, 43), encompassed adjunctive cognitive or physical training (22, 40, 44, 45) to tDCS or adopted inclusion criteria for selecting studies encompassing vascular dementia or other neurological disorders, as well as patients with mild cognitive impairment (46).

Our systematic review tried to bypass the aforementioned limitations and represents an update systematic review of RCTs evaluating the effects of tDCS upon cognition in AD and PD as a stand-alone technique (i.e., without combined cognitive or physical training) compared with sham (i.e., placebo) stimulation.

## Methods

### Search Strategy

**Figure 1:**
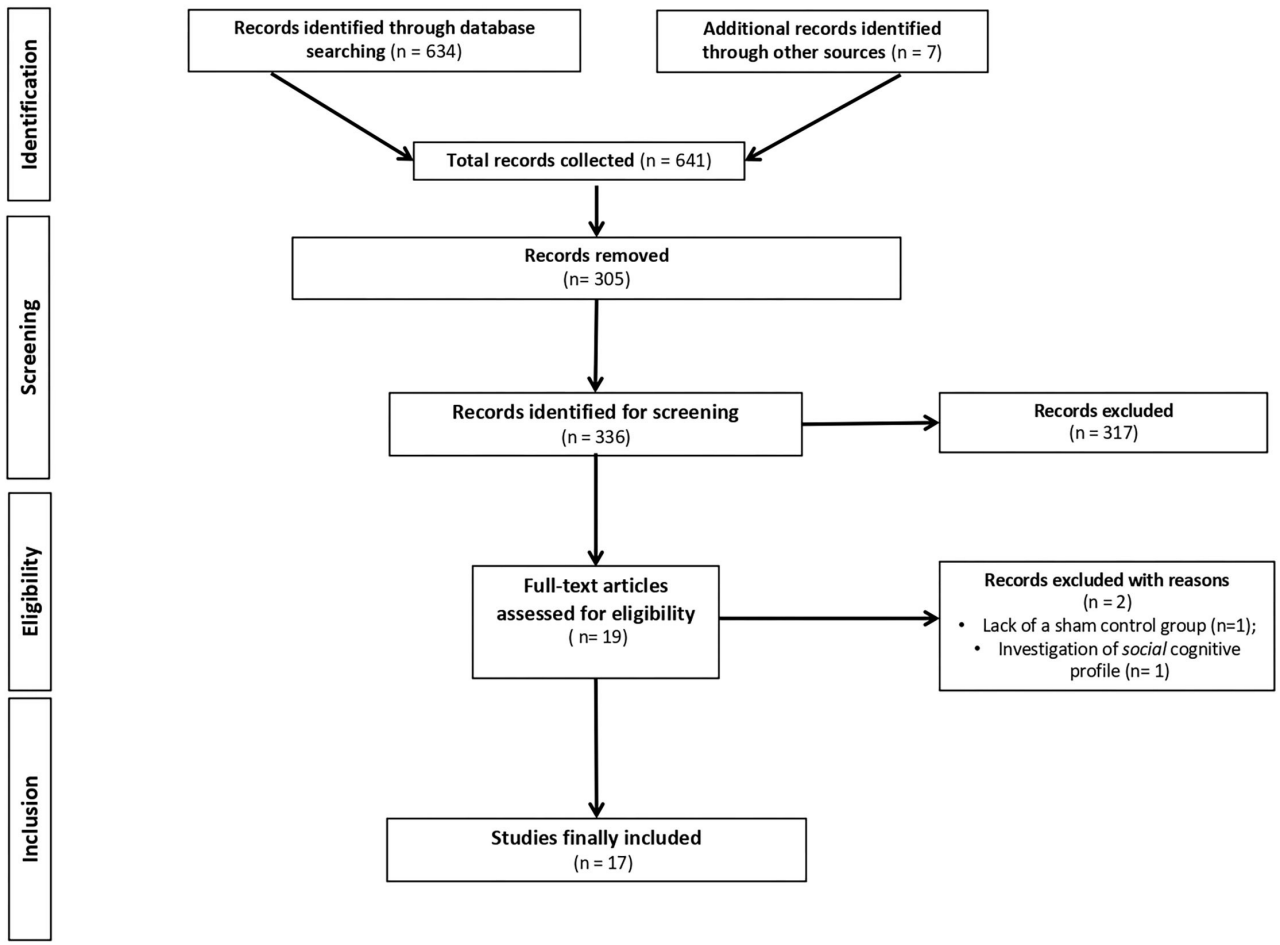
PRISMA (Preferred reporting of systematic reviews and meta-analysis) flowchart of search results.

This update systematic review adheres to the *Preferred Reporting Items for Systematic Reviews and Meta-analyses* (PRISMA) *Statement* (47). PubMed, Web of Science, and Cochrane Library databases were systematically screened for RCTs using the following terms: “Alzheimer's Disease” or “Parkinson's disease” and “transcranial direct current stimulation” and “cognition” or “cognitive abilities” or “cognitive deficits” or “cognitive impairment” (only upper time limit: September 31, 2021). Additional titles were added based on the bibliographies of the relevant issues and through the use of hand search of journals and other pertinent resources. Figure 1 shows the PRISMA flowchart.

### Study Selection Criteria

Studies from the literature search were selected if they met the following criteria: (1) assessing the effects of tDCS on cognitive functioning; (2) including patients with AD or PD selected by recognized international diagnostic criteria, i.e., NINCDS–ADRDA criteria for AD (48) and UK Brain Bank criteria for PD (49), respectively; (3) RCT as study design; (4) measures of cognition as primary or secondary outcomes; (5) presence of sham (i.e., placebo) stimulation. Exclusion criteria encompassed: (1) multicomponent interventions (e.g., tDCS *plus* physical or cognitive training) or high-definition tDCS techniques; (2) other noninvasive brain stimulation techniques (e.g., TMS); (3) studies recruiting individuals with neurological disorders different from AD and PD (i.e., other dementia types or vascular dementia, mild cognitive impairment, stroke, multiple sclerosis, traumatic brain injury, focal brain disorders, etc.) or classified as having mild/major neurocognitive disorder and also psychiatric diseases and other relevant medical conditions that might interfere with cognitive functioning; (4) studies recruiting healthy older adults; (5) animal studies; and (6) manuscripts written in other languages than English.

### Quality of the Studies and Assessment of Risk of Bias Evaluation

Three independent reviewers (DMC, FC, and RC) first evaluated methodological criteria used by RCTs examining tDCS effects upon cognition in AD (Table 1) and PD (Table 2) patients and then assessed the risk of bias according to the *Quality Assessment Tool for Quantitative Studies* (63) developed by the *Effective Public Health Practice Project* (EPHPP) (Tables 3, 4 for AD and PD, respectively). In both the cases, disagreement was discussed until a consensus among reviewers was definitely reached.

**Table 1:** Evaluation of methodological criteria used by RCTs examining tDCS effects for AD.

**References**	**1**	**2**	**3**	**4**	**5**
Ferrucci et al. (50)	+	+	+	+	-
Boggio et al. (51)	+	-	+	+	+/-
Boggio et al. (52)	+	+/-	+	+/-	+
Khedr et al. (53)	+	+	+	+	+
Suemoto et al. (54)	+	+	+	+/-	+
Bystad et al. (20)	+	+	+	+	+/-
Im et al. (12)	+	+	+/-	+	+/-
Khedr et al. (55)	+	+	+	+	+
Gangemi et al. (56)	+	+	+	+	+/-
Gangemi and Fabio (57)	+	+/-	+	-	+

*(1) The diagnosis of AD is based on validated criteria [NINCDS-ADRDA, (48)]; (2) Inclusion and exclusion criteria of the study are specifically described; (3) The study has sufficient statistical power (n ≥ 10 per group); (4) Intervention, measurements and outcomes are fully described; (5) Potential adverse effects are indicated and confounding variables are discussed*.

**Table 2:** Evaluation of methodological criteria used by RCTs examining tDCS effects for PD.

**References**	**1**	**2**	**3**	**4**	**5**
Boggio et al. (10)	+	+	+	+	+
Doruk et al. (58)	-	+/-	+	+	+/-
Ferrucci et al. (59)	-	+/-	-	-	-
Dagan et al. (60)	+	+	+	+	+/-
Bueno et al. (61)	+	+/-	+	+	+
Lau et al. (62)	+	+/-	-	+	+/-
Firouzi et al. (39)	+	+/-	-	+	+/-

*(1) The diagnosis of PD is based on validated criteria [i.e., UK brain bank criteria; (49)]; (2) Inclusion and exclusion criteria of the study are specifically described; (3) The study has sufficient statistical power (n ≥ 10 per group); (4) Intervention, measurements and outcomes are fully described; (5) Potential adverse effects are indicated and confounding variables are discussed*.

**Table 3:** Assessment of risk of bias of the included RCTs pertaining tDCS in AD.

**References**	**Selection bias**	**Study design**	**Confounders**	**Blinding**	**Data collection methods**	**Withdrawals and dropout**	**Overall**
Ferrucci et al. (50)	^***^	^**^	^*^	^**^	^*^	^***^	^**^
Boggio et al. (51)	^***^	^*^	^*^	^*^	^**^	^***^	^**^
Boggio et al. (52)	^***^	^**^	^**^	^**^	^*^	^***^	^**^
Khedr et al. (53)	^***^	^***^	^***^	^**^	^***^	^***^	^***^
Suemoto et al. (54)	^***^	^***^	^*^	^***^	^*^	^***^	^**^
Bystad et al. (20)	^***^	^***^	^*^	^**^	^***^	^**^	^**^
Im et al. (12)	^***^	^***^	^***^	^**^	^***^	^**^	^***^
Khedr et al. (55)	^***^	^**^	^***^	^**^	^***^	^*^	^**^
Gangemi et al. (56)	^***^	^***^	^*^	^**^	^*^	^***^	^**^
Gangemi and Fabio (57)	^***^	^**^	^**^	^**^	^*^	^***^	^**^

* *, Weak quality*;

** *, Moderate quality*;

*** *, Strong quality*.

**Table 4:** Assessment of risk of bias of the included RCTs pertaining tDCS in PD.

**References**	**Selection bias**	**Study design**	**Confounders**	**Blinding**	**Data collection methods**	**Withdrawals and dropout**	**Overall**
Boggio et al. (10)	^***^	^*^	^*^	^**^	^*^	^***^	^**^
Doruk et al. (58)	^**^	^***^	^*^	^**^	^***^	^***^	^**^
Ferrucci et al. (59)	^*^	^**^	^*^	^*^	^*^	^***^	^**^
Dagan et al. (60)	^*^	^**^	^*^	^**^	^***^	^***^	^**^
Lau et al. (62)	^**^	^**^	^**^	^**^	^*^	^***^	^**^
Bueno et al. (61)	^***^	^**^	^*^	^**^	^**^	^***^	^**^
Firouzi et al. (39)	^***^	^**^	^***^	^*^	^**^	^***^	^**^

* *, Weak quality*;

** *, Moderate quality*;

*** *, Strong quality*.

## Results

### Studies Selection, Evaluation, and Report

Initially, 634 records were identified through databases and manual search (Figure 1). After removing duplicates (*n* = 305), we screened the titles and the abstracts of the remaining records and identified 19 articles for a full-text inspection. Two studies (64, 65) were excluded because of different reasons (see Figure 1). Finally, 17 articles were included in our systematic review, 10 pertaining AD (12, 20, 50–57) and 7 pertaining PD (10, 39, 58–62). The evaluation of methodological criteria used was first shown in Tables 1, 2. The assessment of risk of bias (Tables 3, 4) reported that 15 studies were of moderate quality whereas only 2 studies were of strong quality (12, 53).

### Outcomes: tDCS Effects on Cognitive Domains

A summary of the included studies was reported in Tables 5, 6 for patients with AD and for patients with PD, respectively. A total of 9 study designs were parallel ones (12, 20, 53–58, 61) whereas 8 study designs were crossover ones (10, 39, 50–52, 59, 60, 62). More specifically, the *washout* period of the latter studies performing different tDCS stimulations presented in counterbalanced order across participants, substantially varied from 48 h (10, 51) to 71.1 ± 5.8 days (52). A total of 5 studies (52–54, 58, 59) performed a follow-up, varying from 1 week (53, 54, 59) to 2 months (53), with 2 investigations reporting a prolonged tDCS effect upon cognition, particularly on the visual recognition memory (52) and divided attention (58). Furthermore, Gangemi et al. (56) adopted the longest intervention of stimulation (i.e., 10 days a month for 8 months).

**Table 5:** Summary of main results of the selected studies of tDCS in AD.

**References**	**Participants**	**Procedure and brain region/s involved**	**Intervention for active groups**	**Current intensity and electrodes position according to the 10-20 EEG international system**	**Neuropsychological assessment/experimental cognitive tasks**	**Follow-up**	**Main findings of the active group/s at the end of the intervention**
Ferrucci et al. (50)	10 patients Sex: M = 3; F = 7; Mean age: 75.2 ± 7.3; Pharmacotherapy: ChEI	Anodal, cathodal or sham tDCS of the TPC	3 sessions at intervals of 1 week	Anodal or cathodal 1.5 mA current delivered for 15 min bilaterally over the TPC (P3-T5 left side; P4-T6 right side); Cathodal electrode: right deltoid muscle	Word recognition task (modified from Adas-cog); c-attentional cue task (E-Prime computer-controlled Posner paradigm)	-	Word recognition memory improvement after anodal tDCS
Boggio et al. (51)	10 patients Sex: M = 4; F = 6; Mean age: 79.1 ± 8.8 Pharmacotherapy: ChEI (not for all patients), BDZs, antipsychotics, TCAs	Anodal tDCS of the TC, DPFC, or sham tDCS	3 sessions at intervals of 48 h	2 mA intensity delivered for 30 min over the left DLPFC (F3) or left TC (T3); Cathodal electrode: SO	Stroop test; digit span (backward and forward); computer-based recognition memory task	-	Visual recognition memory improvement both after temporal and prefrontal tDCS
Boggio et al. (52)	15 patients Sex: M = 8; F = 7 Mean age: 71.1 ± 5.8; Pharmacotherapy: not reported	Anodal or sham tDCS of the TC	5 consecutive days sessions	2 mA current delivered for 30 min bilaterally (T3, T4) Cathodal electrode: right deltoid muscle	MMSE, Adas-Cog, c-VRT, c-VAT	At 1 week and at 1 month	Visual recognition memory improvement and maintenance 4 weeks after the intervention
Khedr et al. (53)	34 patients Sex: M = 19; F = 15 Mean age of anodal group: 68.5 ± 7.2 Mean age of cathodal group: 70.7 ± 5.4 Mean age of sham group: 67.3 ± 5.9 Pharmacotherapy: no patients took cholinomimetics, antidepressants, neuroleptics, sedative-hypnotics drugs for at least 1 week before assessment	Anodal, cathodal or sham tDCS of the DLPFC	10 daily sessions	2 mA intensity delivered for 25 min Anodal/sham group: anodal electrode over the left DLPFC (F3), cathodal electrode: contralateral SO Cathodal group: cathodal electrode over the left DLPFC (F3), and anodal electrode over the contralateral SO	MMSE, WAIS-III	At 1 and 2 months	Global cognition (MMSE) improvement both after anodal and cathodal tDCS
Suemoto et al. (54)	40 patients Sex: M = 12; F = 28 Mean age of anodal group: 79.4 ± 7.1 Mean age of sham group: 81.6 ± 8.0 Pharmacotherapy: ChEI	Anodal or sham tDCS of the DLPFC	6 sessions over a period of 2 weeks	2 mA intensity delivered for 20 min Anode electrode: left DLPFC (F3) Cathodal electrode: contralateral SO	Adas-Cog	At 1 week	No improvement
Bystad et al. (20)	25 patients Sex: M = 14; F = 11 Mean age of anodal group: 70.0 ± 8.0 Mean age of sham group: 75.0 ± 8.7 Pharmacotherapy: ChEI	Anodal or sham tDCS of the TC	6 sessions for 10 days	2 mA intensity delivered for 30 min Anode electrode: left temporal lobe (T3) Cathodal electrode: right frontal lobe (Fp2)	CVLT, MMSE, CDT, TMT	-	No improvement
Im et al. (12)	18 patients Sex: M = 3; F = 15 Mean age of anodal group: 71.9 ± 9.2 Mean age of sham group: 74.9 ± 5.0 Pharmacotherapy: ChEI	Anodal or sham tDCS of the DLPFC	Daily sessions for 6 months	2 mA intensity delivered for 30 min Anode electrode: left DLPFC (F3) Cathodal electrode: right DLPFC (F4)	MMSE, Digit span forward and backward, BNT, RCFT, CDT, SVLT, contrasting program, Go-No-Go test, COWAT, Stroop test	-	Improvement of global cognition (MMSE) and language (BNT); preventive decrease of executive functions
Khedr et al. (55)	44 patients Sex: M = 26; F = 18 Mean age of anodal group: 64.2 ± 3.64 Mean age of sham group: 65.2 ± 4.5 Pharmacotherapy: Memantine and piracetam	Anodal or sham tDCS of the right and left temporal lobe	5 sessions/wk for 2 consecutive weeks	2 mA intensity delivered for 20 min for each side Anode electrode: right TL/left TL (T3-P3/T4-P4) Cathodal electrode: deltoid muscle of the left arm	Modified-MMSE, CDT, Montreal Cognitive Scale	-	A significant improvement in the total score of each cognitive rating scale in the real group
Gangemi et al. (56)	Study 1 26 patients Sex: M = 10; F = 16 Mean age of anodal group: 67.25 ± 2.8 Mean age of sham group: 69 ± 6.1 Study 2 18 patients Sex: M = 5; F = 13 Mean age of anodal group: 68.5 ± 2.8 Mean age of sham group: 68.7 ± 3.1 Pharmacotherapy: ChEI	Anodal or sham tDCS of the left frontotemporal lobe	Study 1: daily sessions for 10 consecutive days Study 2: daily sessions for 10 consecutive days each month for 8 months	2 mA intensity delivered for 20 min Study 1 Anode electrode: left frontotemporal lobe (F7-T3); Cathodal electrode: right frontal lobe (Fp2).	MMSE MODA	-	tDCS intervention was effective both in the short- and the long-term to slow down the progression of AD on temporal and personal orientation, attention, calculation, and recall
Gangemi and Fabio (57)	26 patients Sex: M = 14; F = 12 Mean age of anodal group: 72 ± 4.4 Mean age of sham group: 75 ± 4.4 Pharmacotherapy: ChEI	Anodal or sham tDCS of the left frontotemporal cortex	10 sessions	Anode electrode: DLPFC (F3-F7), and left (F7) Cathodal electrode: right SO	MODA subscales (temporal orientation, spatial orientation, personal orientation, family orientation, autonomy, reversal learning, verbal intelligence, story test, words production, token test, digital agnosia, constructive apraxia, Street test, attentional test)	-	Improvements of temporal orientation, spatial orientation, reversal learning, verbal intelligence, story test, word production and attention

*AD, Alzheimer's disease; ChEI, cholinesterase inhibitors; TPC, Temporoparietal Cortex; DBZs, benzodiazepines; TCAs, Tricyclic antidepressants, SSRIs, Selective serotonin reuptake inhibitors; TC, Temporal Cortex; DLPFC, Dorsolateral Prefrontal Cortex; MMSE, Mini Mental State Examination; Adas-cog, Alzheimer's Disease Assessment Scale-cognitive subscale; c-VRT, computerized Visual Recognition Task; WAIS-III, Wechsler Adult Intelligence Scale Third Edition; CVLT, California Verbal Learning Test; c-VRT, computerized Visual Recognition Task; WAIS-III, Wechsler Adult Intelligence Scale Third Edition; CDT, Clock Drawing Test; TMT, Trail Making Test; BNT, Boston Naming Test; RCFT, Rey Complex Figure Test; SVLT, Seoul Verbal Learning Test; COWAT, Controlled Oral Word Association Test; TL, Temporal lobe; MODA, Milan Overall Dementia Assessment; HD-tDCS, High-definition tDCS*.

**Table 6:** Summary of main results of the selected studies of tDCS in PD.

**Study**	**Participants**	**Procedure and brain region/s involved**	**Intervention for active groups**	**Current intensity and electrodes position according to the 10-20 EEG international system**	**Neuropsychological assessment/experimental cognitive tasks**	**Follow-up**	**Main findings of the active group/s at the end of the intervention**
Boggio et al. (10)	18 patients (9 patients for each experiment) Sex: M = 12; F = 6 Mean age: (Experiment 1): 59.2 ± 9.9 Mena age: (Experiment 2): 61.0 ± 12.1; Pharmacotherapy: patients were withdrawn from antiparkinsonian drugs for 12 h	Anodal tDCS of the DLPFC or PMC and sham tDCS	3 sessions at intervals of 48 h	1 mA or 2 mA intensity delivered for 20 min Anode electrode: left DLPFC (F3) or PMC (C3); Cathodal electrode: contralateral RSO	Three-back letter WM paradigm (during tDCS)	-	WM improvement after anodal tDCS of the DLPFC
Doruk et al. (58)	18 patients Sex: M = 7; F = 9 Mean age: 61.0 ± 8.0 Pharmacotherapy: Stable medication (L-dopa) regimen 1 month prior the study	Anodal tDCS of the DLPFC and sham tDCS	10 session over 2 weeks	2 mA intensity delivered for 20 min; Anode electrode: left DLPFC (F3) or right DLPFC (F4) Cathodal electrode: contralateral SO	TMT (Part A and B), WCST, PCL, WMT, Stroop Test	At 1 month	Prolonged improvement of divided attention (TMT Part B)
Ferrucci et al. (59)	9 patients Sex: M = 5; F = 4 Mean age: 74.3 ± 7.9; Pharmacotherapy: Stable medication (L-dopa) regimen 2 months prior the study	Anodal cerebellar tDCS, anodal MC and sham tDCS	5 consecutive session in a week at intervals of 1 month	2 mA intensity delivered for 20 min; Anode electrode: over the right and left cerebellum/motor cortex bilaterally (C3 and C4); Cathodal electrode: right deltoid muscle	Word recall task, VAT, SRTT	At 1 week and 1 month	No improvement
Dagan et al. (60)	20 patients Sex: M = 17; F = 3 Mean age: 68.8 ± 6.8 Pharmacotherapy: Stable medication (L-dopa) regimen 1 month prior to the study	Anodal tDCS of the PMC and DLPFC simultaneously, PMC only and sham tDCS	3 sessions at intervals of 48 h	1,5 mA intensity delivered for 20 min; Anode electrode: Medial motor cortex (CZ) and left DLPFC (F3)/medial motor cortex (CZ); Cathodal electrode: not reported	Catch-Game, Go-No-Go task, Stroop Test, Staged Information Processing Speed and NeuroTrax	-	Decrease of sensitivity to interference (Stroop Test) after combined stimulation
Lau et al. (62)	10 patients Sex: M = 5; F = 5 Mean age: 62.7 ± 6.6 Pharmacotherapy: antiparkinsonian medications	Anodal or sham tDCS of the DLPFC	2 sessions with an interval of 2 weeks	2 mA intensity delivered for 20 min; Anode Electrode: left DLPFC (F3); Cathodal electrode: contralateral SO	MMSE, a visual working memory task and a go/no-go test	-	tDCS is ineffective in improving cognitive tasks administered
Bueno et al. (61)	20 patients Sex: M = 8; F = 12 Mean age: 64.45 ± 8.98 Pharmacotherapy: antiparkinsonian medications	Anodal or sham tDCS of the DLPFC	2 session with a one-week interval	2 mA intensity delivered for 20 min; Anode electrode: left DLPFC (F3); Cathodal electrode: right OFC	TMT, Stroop Test, Verbal Fluency	-	Improvements in verbal fluency and sensitivity to interference
Firouzi et al. (39)	11 patients Sex: M = 8; F = 3; Mean age: 77.1 ± 4.88 Pharmacotherapy: Levodopa medication (stable regimen)	Anodal/sham tDCS intervention during the SRT task.	4 sessions with an interval of 1 week between the first and the second session and between the third and the fourth and 3 weeks between the second and the third ones.	2 mA intensity delivered for 20 min during the SRT task. Anode electrode: C3 or C4, Cathodal electrode: Fp1 when the active electrode was on C4; on Fp2 when the active electrode was on C3.	SCOPA-COG, MMSE SRT task	-	Positive effects on implicit motor sequence learning (IMSL)

*DLPFC, dorsolateral prefrontal cortex; PMC, primary motor cortex; WM, working memory; TMT, Trail Making Test; WCST, Wisconsin Card Sorting Test; PCL, Probabilistic Classification Learning; WMT, Working Memory task; MC, motor cortex; VAT, Visual Attention Task; SRTT, Serial Reaction Time Task; PD-MCI, Parkinson's disease-Mild Cognitive Impairment*.

Remarkably, participants of AD recruited from the studies were different in terms of global cognition at baseline. A range of Mini Mental State Examination (MMSE) scores was present among studies, with that of (54) (i.e., 15.0 ± 3.1 for the tDCS group and 15.2 ± 2.6 for the sham group) reporting the lowest ones. Such a discrepancy was not revealed for the PD selected studies.

In patients with AD, recognition memory—both verbal and visual one—improved at different current intensities, stimulation duration, and number of sessions (i.e., from 1.5 to 2 mA, from 15 to 30 min, from 3 to 5 sessions, respectively) by tDCS of the temporal cortex (50–52) while a clear-cut effect on the global cognition was obtained after a 2 mA stimulation for 25 of 30 min of the left DLPFC (12), both on the anodal and cathodal modality (53) or anodal stimulation of the frontotemporal cortex (56, 57). Visuoconstructive ability (55) and language abilities (i.e., naming) (12) seemed to ameliorate after daily sessions (2 mA for 20/30 min) of tDCS, too.

In patients with PD, executive efficiency was enhanced either thanks to the stimulation of DLPFC as a single brain area through variable sessions of treatment (i.e., 1–10 sessions) at 1–2 mA of 20-min current stimulation (10, 58, 61) or thanks to the combined stimulation of DLPFC and primary motor cortex (PMC) at 1.5 mA after a 3-session intervention of 30 min (60). Finally, beneficial effects of anodal tDCS over the primary motor cortex were found in relation to IMSL in such patients (39) after 1 week from the intervention (i.e., 2 mA intensity for 20 min per session during the cognitive task). In four cases, no cognitive improvement was revealed after tDCS intervention (20, 54, 59, 62).

Transcranial DCS was well tolerated by the patients even if some side effects were sometimes reported (i.e., tingling, sleepiness, mild headache, neck pain, skin redness, scalp pain, scalp burning, somnolence, and trouble concentrating) [e.g., (53, 54, 58)].

## Discussion

This systematic review aimed to provide a comprehensive overview of the current knowledge about the effects of tDCS stimulation upon cognition for patients with AD and PD when compared with sham (*placebo*) stimulation. Transcranial DC stimulation seems to ameliorate cognitive vitality of patients in relation to global cognition and recognition memory in AD and divided attention, verbal fluency, and reduction of sensitivity to interference in PD, respectively. From a neuropsychological point of view, criticism remains about potential usefulness of tDCS for working memory, processing speed and visual attention, visuospatial abilities and verbal learning performances while initial proof arises about language improvement after tDCS.

Heterogeneity of the patients (i.e., age, disease onset, severity and duration, premorbid level of functioning), tDCS delivery settings (i.e., clinics, hospitals, and home treatment), concomitant pharmacological therapy and concurrent psychopathological symptoms, particularly depression and apathy not routinely evaluated except for some investigations represent confounding variables that make difficult to compare among studies [cf. (66)].

In four cases (20, 54, 59, 62), we reported the lack of results about cognition improvement after tDCS. In our opinion, it should be because of the use of the neuropsychological assessment/experimental tasks adopted for the evaluation of cognitive functions associated to the stimulated brain areas and/or small sample size.

Some researchers (12, 53, 54) stimulated the DLPFC in patients with AD, as a brain area critically associated with working memory and to a repertory of the frontal abilities, including planning, abstract reasoning, mental flexibility, and attentional set shifting. A recent investigation using [11C]-raclopride positron emission tomography demonstrated that tDCS of the DLPFC enhance attention system and executive functioning because of an increased release of dopamine neurotransmitter (67) in healthy males, probably allowing a more accurate performances on cognitive tests requiring an additional recruitment of attentional resources and executive control.

Other researchers (10, 58, 60) investigated the activation of the same brain area in patients with PD with encouraging findings, given that it has been related to executive deficits because of the dopaminergic dysfunction of the fronto-striatal network and to top–down attentional deficits due to alterations of the cholinergic fronto-parietal circuits, commonly reported in these patients (68). Remarkably, the enhancement of locomotor skills of patients with PD may benefit from executive efficiency (69) too. It has also been suggested that a possible beneficial effect of tDCS specific stimulation for patients with PD could be the induction of dopamine release in the caudate nucleus *via* the glutamatergic corticostriatal pathway, as shown in animal studies (70). Transcranial DC stimulation might also have a neuroprotective role in PD, by reducing the oxidative damage of dopamine neurons and by modulating functional connectivity of the corticostriatal and thalamocortical circuits of the human brain (40).

The selected studies of Boggio et al. (51, 52) and Bystad et al. (20) stimulated the medial temporal cortex in patients with AD, as a brain area critically associated with different memory performances [cf. (71, 72)]. Alterations of the medial temporal lobe which might influence visual recognition memory are well recognized in patients with AD (73, 74). Patients with AD also report a selective hypoactivation of the temporoparietal cortex (TPC) that is normally involved in word recognition memory tasks (75). Transcranial tDCS of the TPC seems to enhance such a memory process (50), too.

Despite the difficulty of drawing definitive conclusions, results from the RCTs globally show a tendency toward positive effects of tDCS for patients with AD and PD, however, it is less clear which stimulation procedure leads to the best results. Evidence-based guidelines developed by Lefaucheur et al. (76) on the therapeutic use of tDCS reported no recommendation for the efficacy of specific tDCS parameters (i.e., electrodes placement, number and timing of sessions and duration, intensity and time of stimulation), by concluding that the optimization of tDCS protocols should be better addressed in the next future to offer a more pronounced therapeutic effect also in case of patients with AD and PD. Recently, given that tDCS has showing promising clinical results, a team of experts in conducting systematic reviews of the clinical trials have concluded that such rehabilitation technique is probably effective in PD, both for motor and cognitive aspects (77).

Some studies have shown that connections between different areas of the AD brain are impaired at specific time points, and that stimulation of other brain areas not primarily associated with commonly impaired cognitive functioning could yield promising results. A fundamental aspect of AD pathophysiology is based on the dysfunction of long-range cortical networks (78). As an illustration, not only the hippocampus and the associative cerebral cortices are involved in memory processes but also the posterior parietal cortex (PPC) exerts a key role for attentional resources in supporting memory processes (79), i.e., notoriously damaged in AD. Recently, a notable investigation has shown that mechanisms of cerebellar-cortical plasticity are impaired in AD (80) too. Given its role in the higher cognitive functions, new potential therapeutic strategies should be also built up in the next future to modulate neural activity in the cerebellum.

Focus on the neurophysiological aspects of other neurostimulation techniques, such as TMS/transcranial alternating current stimulation (tACS) and EEG activity, may offer supplementary information able to deeply investigate brain circuitry modulation. For example, mechanisms of cortical plasticity have been investigated in patients with AD by TMS protocols, such as theta brust stimulation (TBS) showing a clear impairment of long-term potentiation (LTP) cortical-like plasticity and a relative sparing of long-term depression (LTD) mechanisms in AD (78). There is also evidence that spike-timing-dependent plasticity (STDP) is compromised in AD, as revealed by studies adopting paired associates stimulation (PAS) protocols (81). Similarly, some interesting studies using cerebellar continuous TBS have reported promising results that may help identifying specific neurophysiological phenotypes as that shown by a group of patients clinically diagnosed as PD with normal dopaminergic functional imaging defined as SWEED (Scans Without Evidence of Dopaminergic Deficit). Patients with SWEED present with a mild impairment in cerebello-thalamo-cortical circuit and this neurophysiological phenotype differs from the that observed in PD and dystonic patients, suggesting a distinct involvement of this pathway in the pathophysiology of disorders (82).

These observations could lead researcher to implement neurostimulation techniques exploring different sites of stimulation or to even consider multisites techniques that could give more insight on the correct parameters to be used. A recent area of interest is represented by an implementation of tDCS technique, namely, high-definition tDCS (i.e., HD-tDCS), a novel approach that uses smaller electrodes whose configuration can be optimized for targeting specific brain regions (83). Such a technique offers some advantages then conventional method, as follows: (i) it can stimulate more precisely a target cortical region; (ii) aftereffects last at least 30 min longer than those obtained with conventional tDCS; (iii) it potentially reduces the likelihood of side effects; (iv) it determines less discomfort and improves applicability in the elderly (84).

Given the relatively minor neurodegenerative changes, tDCS appears to be more promising in early phases of the disease, i.e., MCI due to AD and in the Parkinson's disease mild cognitive impairment (PD-MCI), as confirmed by preliminary investigations also when it is delivered alongside cognitive or physical training (85–88). Accordingly, it has already been documented how tDCS stimulation is less effective in patients with AD in the advanced stages of the disease (43, 89). Moreover, tDCS can modulate brain activity in a manner similar to TMS with the advantages of being easily applied and substantially safe. Our review confirms that tDCS is well-tolerated by the patients with slight side effects not frequently reported (i.e., tingling, sleepiness, mild headache, neck pain, skin redness, scalp pain, scalp burning, somnolence, and trouble concentrating). Transcranial DC stimulation is also reliably blinded by placebo (i.e., sham stimulation) in the clinical settings.

Despite emerging evidences, the larger RCTs are welcome in the next future for replicating preliminary results on patients with AD and PD and for measuring the effects on different outcomes beside cognition (i.e., psychopathological dimensions such as depression and/or apathy, health-related quality-of-life, personal and instrumental autonomy and also motor functioning for patients with PD) allowing researchers to depict a more comprehensive analysis of tDCS potential.

Cortical plasticity and connectivity result to be impaired in the neurodegenerative conditions and neurophysiological findings could provide more robust evidence about the implementation of tDCS protocols for these diseases. The optimization of tDCS protocols should also start from early response of the patient to the treatment. Future tDCS studies would also take advantage of computational models to ensure a calibration of the stimulation technique on specificity of the patient (90). Finally, tDCS should be widely delivered to patients with neurological disorders as an *at-home* rehabilitation strategy under supervision of therapists, in order to improve personalized medicine purposes [cf. (91–93)].

## Data Availability Statement

The original contributions presented in the study are included in the article/supplementary material, further inquiries can be directed to the corresponding author.

## Author Contributions

DMC made the most substantial contribution in data conception, knowledge synthesis, acquisition, analysis, interpretation of data, and as well as manuscript writing. FC and RC were involved in checking for quality of the studies and risk of bias evaluation. UB and GC revised the paper for intellectual content. All the authors approved the final version of the manuscript.

## Conflict of Interest

The authors declare that the research was conducted in the absence of any commercial or financial relationships that could be construed as a potential conflict of interest.

## Publisher's Note

All claims expressed in this article are solely those of the authors and do not necessarily represent those of their affiliated organizations, or those of the publisher, the editors and the reviewers. Any product that may be evaluated in this article, or claim that may be made by its manufacturer, is not guaranteed or endorsed by the publisher.
